# Supplementary figures and images for: Cellular Requirements for Building a Retinal Neuropil
Source: Cell Rep. 2013 Feb 21;3(2):282–90. doi: 10.1016/j.celrep.2013.01.020 (PMC3607253; doi:10.1016/j.celrep.2013.01.020)

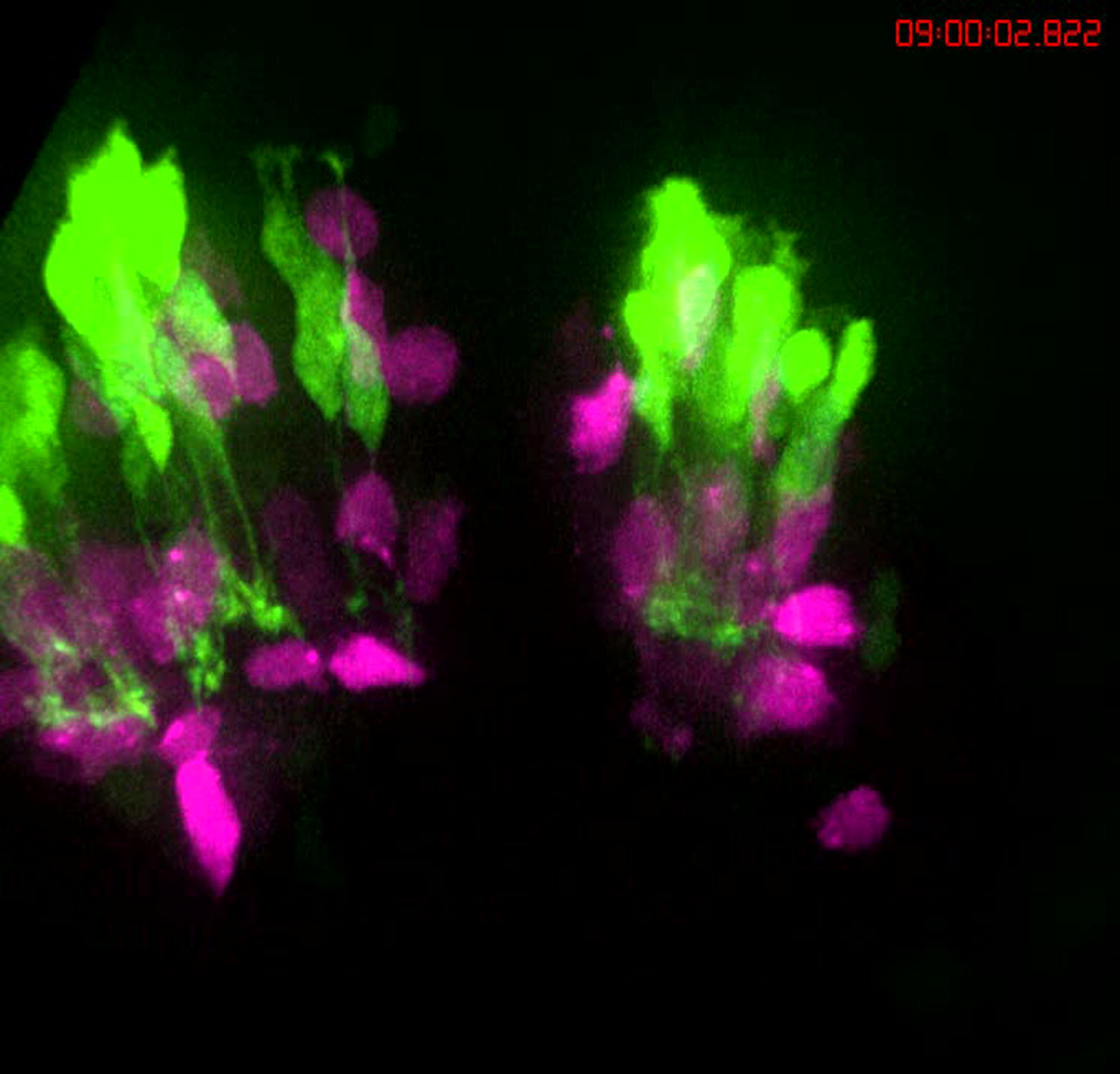

Supplement: Movie S2. BC Axons Are among the Earliest Invaders of the Nascent IPL, Related to Figure 1 — Time-lapse spinning disk confocal imaging of mosaic retinas containing vsx1:GFP-expressing BCs (green) and ptf1a:DsRed-expressing ACs (magenta) in an unlabeled host retina. At the onset of the imaging session, ptf1a:DsRed expressing ACs and HCs have migrated to the AC layer. However, a separation between the dACs and ACs is not apparent. Over time, the BC axons appear and begin to stratify within the ptf1a-expressing cells (arrowheads). As these BC axons elaborate, the ACs are separated into displaced and nondisplaced populations, which are parted by the expanding IPL. Images are confocal reconstructions. Time shown in hr:min. Imaging begins at ∼40 hpf. [file mmc2.jpg]

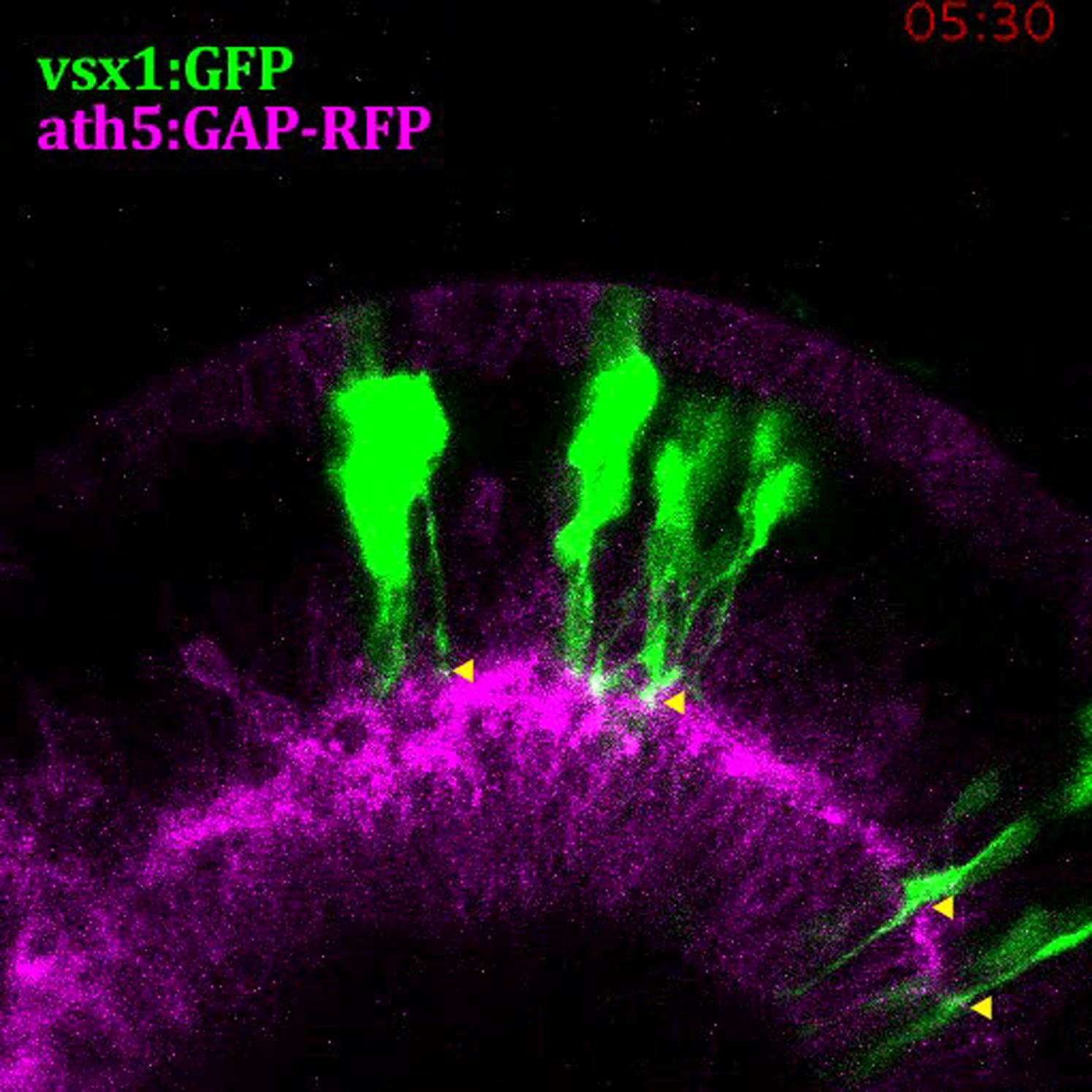

Supplement: Movie S3. BC Axons Arrive Coincident with ath5:GAP-RFP-Labeled Neurites in the IPL, Related to Figure 1 — Time-lapse confocal imaging of mosaic retinas vsx1:GFP-expressing BCs (green) in a host retina where RGCs and ACs are labeled by ath5:GAP-RFP (magenta). GFP-expressing BC axons (arrowheads) accumulate coincidentally with the appearance of the IPL, as shown by ath5:GAP-RFP-labeled RGCs and ACs. Images represent maximum intensity projections of nine confocal slices. Time shown in hr:min. Imaging begins at ∼40 hpf. [file mmc3.jpg]

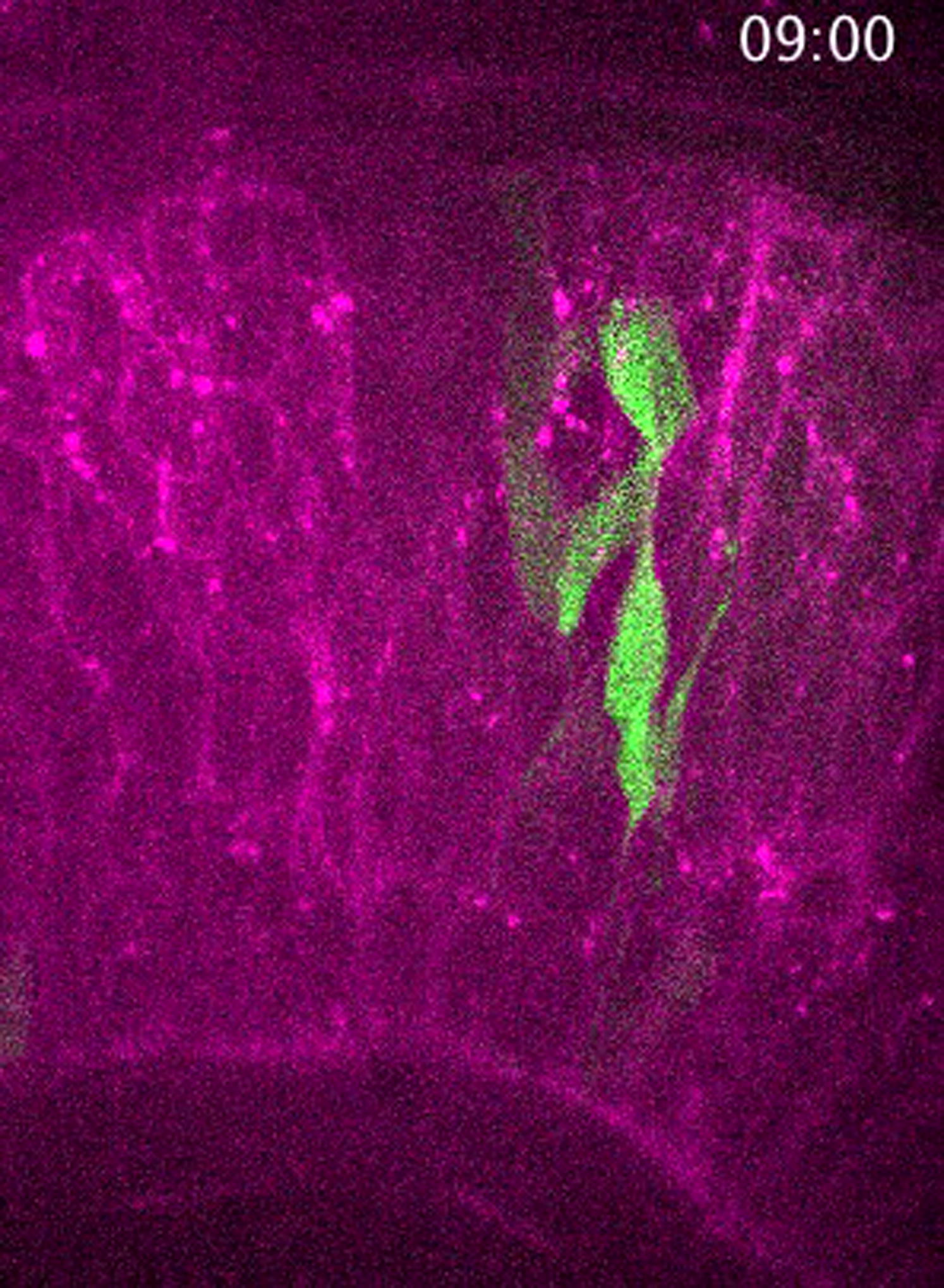

Supplement: Movie S4. BC Axons Accumulate in the Nascent IPL at Approximately the Same Time as Processes from Partner Neurons, Related to Results and Figure S1 — Time-lapse confocal imaging of vsx1:GFP-expressing BCs within a membrane-mCherry-labeled retina. The IPL becomes visible in the mCherry channel as a wavy band, which thickens and condenses into the IPL. The GFP-expressing BC axons are visible within the nascent IPL (arrowheads) and elaborate as the IPL matures. Images are of a single confocal slice. Time shown in hr:min. Imaging begins at ∼40 hpf. [file mmc4.jpg]
