# Supplementary material for: Cellular Requirements for Building a Retinal Neuropil
Source: Cell Rep. 2013 Feb 21;3(2):282–90. doi: 10.1016/j.celrep.2013.01.020 (PMC3607253; doi:10.1016/j.celrep.2013.01.020)
Supplement: Document S1. Article plus Supplemental Information [file mmc5.pdf]

Cell PRESS
Open ACCESS
Cell Reports
Report

Cellular Requirements for Building a Retinal Neuropil

Owen Randlett,[1,3,4] Ryan B. MacDonald,[1,3] Takeshi Yoshimatsu,[2] Alexandra D. Almeida,[1] Sachihiro C. Suzuki,[2] Rachel O. Wong,[2] and William A. Harris[1,*]
[1]Department of Physiology, Development and Neuroscience, University of Cambridge, Cambridge CB2 3DY, UK
[2]Department of Biological Structure, University of Washington, 1959 NE Pacific Street, Seattle, WA 98195, USA
[3]These authors contributed equally to this work
[4]Present address: Department of Molecular and Cellular Biology, Harvard University, 16 Divinity Avenue, Cambridge, MA 02138, USA
*Correspondence: harris@mole.bio.cam.ac.uk
http://dx.doi.org/10.1016/j.celrep.2013.01.020

SUMMARY

How synaptic neuropil is formed within the CNS is poorly understood. The retinal inner plexiform layer (IPL) is positioned between the cell bodies of amacrine cells (ACs) and retinal ganglion cells (RGCs). It consists of bipolar cell (BC) axon terminals that synapse on the dendrites of ACs and RGCs intermingled with projections from Müller glia (MG). We examined whether any of these cellular processes are specifically required for the formation of the IPL. Using genetic and pharmacological strategies, we eliminated RGCs, ACs, and MG individually or in combination. Even in the absence of all of these partner cells, an IPL-like neuropil consisting of only BC axon terminals still forms, complete with presynaptic specializations and sublaminar organization. Previous studies have shown that an IPL can form in the complete absence of BCs; therefore, we conclude that neither presynaptic nor postsynaptic processes are individually essential for the formation of this synaptic neuropil.

INTRODUCTION

Synaptic neuropil is a major component of nervous systems, yet how it forms in specific places is not understood. It consists of intermingled axonal, dendritic, and glial processes, which form a dense array of specialized cellular connections. In many brain regions, neuropils form discrete layers beside or between collections of highly connected neurons. In the vertebrate retina (Figure 1A) there are two major layers of neuropil: the inner plexiform layer (IPL) and the outer plexiform layer (OPL). These neuropils, which consist of sets of cell-type-specific synapses, are completely devoid of cell bodies and thus are ideal models for studying the formation of synaptic neuropil. The IPL is where bipolar cell (BC) axons synapse onto the dendritic processes of retinal ganglion cells (RGCs) and the neurites of amacrine cells (ACs). The retina contains a single type of intrinsic glial cell, called Müller glia (MG). MG span the retina and send extensive processes into the plexiform layers (Figure 1A). Within the IPL, dendritic and axonal processes stratify within up to ten discrete sublaminae. This segregation relies on adhesive and repulsive guidance cues present in the IPL to guide them to their correct partner neurons (Matsuoka et al., 2011a, 2011b; Yamagata and Sanes, 2008, 2012; Yamagata et al., 2002). It is thought that for such interactions to occur, cues must be expressed either by synaptic partners or by other neurons or glia projecting within the neuropil (Timofeev et al., 2012; Matsuoka et al., 2011a). Therefore, it is important to identify the cell types that are critical for controlling the development of the neuropil layers.

One might hypothesize that the earliest-born cells, the RGCs, could organize a pre-IPL scaffold via their apically emerging dendrites. However, in *ath5 (atoh7)* mutants, RGCs are absent, yet the IPL still forms (Kay et al., 2004). Similarly, BCs appear to be unnecessary for IPL formation, as the IPL still forms in *Chx10; p27* (Green et al., 2003) and *Math3; Mash1* (Tomita et al., 2000) double-mutant mice, which completely lack BCs. It has been suggested that ACs establish the IPL (Huberman et al., 2010; Kay et al., 2004). This suggestion was bolstered by a recent study that showed that extra misplaced IPLs formed when ACs failed to polarize their dendritic processes properly due to a loss of the protocadherin Fat3 (Deans et al., 2011). This work established the sufficiency of ACs for IPL formation, but whether they are necessary for such formation has not been yet tested. MG are also strong candidates for establishing the IPL, because retinal reaggregates form recognizable cell and plexiform layers when grown in the presence of a monolayer of MG (Willbold et al., 2000), but are disorganized and structurally inverted in the absence of MG (Layer et al., 1998).

To determine whether any particular component cells are essential for IPL-like neuropil formation, we selectively removed ACs, MG, and RGCs as individual cell types or in combination using mutants, morpholinos, and pharmacological inhibitors. Surprisingly, an IPL-like neuropil still formed in cellularly simplified retinas consisting of only BCs and photoreceptors (PRs). Remarkably, in this presynaptic-only neuropil, BC axons could still make presynaptic structures and display sublaminar organization of their axonal terminals. Together with previous findings, our results indicate that no single retinal cell type is critical for the formation of an IPL-like neuropil, and suggest that neuropil formation in the vertebrate CNS may result from the coordinated action of multiple autonomously stratifying cell types.

CrossMark

Cell PRESS Open ACCESS

RESULTS

BC Basal Processes Retract from the Basal Surface of the Retina to Stratify Early within the Nascent IPL

To identify when BC axons first begin to enter the IPL, we labeled BCs either by *vsx1:GFP* or individually by the MAZe transgene (Collins et al., 2010), and imaged them by time-lapse confocal microscopy. BCs at early stages of their stratification usually have a thin distal basal process that extends to the basal lamina (Figures 1B and S1A; Movie S1). Later these distal processes retract, and BC axons branch within the IPL (arrows). This retraction is similar to that previously described for mouse BCs (Morgan et al., 2006), although this process happens much faster in zebrafish, taking 01:48 ± 00:13 (hr:min, mean ± SEM, n = 10 cells from four retinas) compared with 1 week in mice. The Kif5c560-based axon reporter (Distel et al., 2010; Jacobson et al., 2006; Randlett et al., 2011) labels these BC processes during the retraction and branching phase (Figure S1B, arrowheads) suggesting that these are indeed axonal processes.

If AC dendrites are critical for IPL formation, one would expect them to arborize into the nascent IPL before the BC axons do. To test this, we transplanted cells from transgenic donors containing the *vsx1:GFP* (to label BCs) and *ptf1a:DsRed* (to visualize all ACs and horizontal cells [HCs]) transgenes into unlabeled hosts (Godinho et al., 2005; Jusuf and Harris, 2009; Vitorino et al., 2009). These studies showed that BC axons began to collect among the cell bodies of differentiating ACs (arrows in Figure 1C; Movie S2) and appeared to part the displaced ACs (dACs) and normal ACs. This result supports the idea that BC axons arrive relatively early within the forming IPL. A similar imaging strategy, using the *ath5:GAP-RFP* transgenic to label RGCs and many ACs, demonstrated that BC axon terminals did not follow the emergence of an RFP-labeled plexus. Instead, the first BC axon branches and the RFP-labeled plexus became visible in the prospective IPL at approximately the same time (arrows in Figure 1D; Movie S3). Finally, we imaged the developing IPL using a fluorescent membrane marker expressed by all retinal cells. This allowed us to visualize BC axonal branches in an environment where all membranes were labeled (Figure S1C; Movie S4). Again, BC axons were visible within the earliest signs of IPL structure (arrows), indicating that BC axons are among the earliest colonizers of the IPL.

ACs Are Not Required for IPL Formation

Because AC dendrites do not obviously lead BC axons with respect to the time of arborization in the IPL, we wondered whether ACs are necessary for IPL formation. Ptf1a is a transcription factor that is expressed by all ACs and HCs in the zebrafish retina, and its disruption causes the respecification of these inhibitory neurons into excitatory ones (Jusuf et al., 2011; Jusuf and Harris, 2009). Ptf1a morpholinos alone do not completely remove all ACs (Jusuf et al., 2011), so we made use of a tilling mutant from the Zebrafish Mutation Project (*ptf1a*^{sa126}). The *ptf1a*^{sa126} mutant allele is a nonsense mutation that results in a truncation within the loop domain (Figure S2A). The *ptf1a*$^{-/-}$ embryos did not have an obvious morphological phenotype. Although they were markedly reduced in numbers, a substantial number of 5E11- and HuC/D-positive ACs remained in the *ptf1a*$^{-/-}$ embryos, indicating that the *ptf1a*^{sa126} allele is not a null mutation (Figures S2B and S2C). However, when two translation-blocking morpholino oligonucleotides (MO; Jusuf et al., 2011) were coinjected into the *ptf1a*$^{-/-}$ mutants with the *p53* MO to reduce nonspecific apoptotic effects (Robu et al., 2007), nearly all ACs were eliminated. Less than 1% of the HuC/D-positive cells in the AC layer remained throughout the retina (mean ± SEM for wild-type [WT] = 3482 ± 614 ACs, *ptf1a*$^{-/-}$*; ptf1aMOs* = 33 ± 15.7 cells, n = 10 retinas at 72 hr postfertilization [hpf]), and there were large stretches of retina with no HuC/D-positive cell bodies or 5E11 staining (Figures S2i and S2ii). Despite the lack of ACs, phalloidin staining indicated that a robust F-actin-rich, IPL-like neuropil was still able to form in these retinas, although it was clearly thinner than the normal IPL (Figures 2A and 2B).

BC Partner Neurons Are Not Required for Neuropil Formation

Because neither of the two postsynaptic partners of BCs on their own appeared to be needed to form an IPL, we wondered whether they might act redundantly in this regard. If so, eliminating both RGCs and ACs simultaneously might lead to a failure of IPL formation. The *ptf1a*$^{-/-}$*;ptf1aMOs* treatment, along with the *ath5/lakritz* mutant (in which RGCs are absent; Kay et al., 2001), provided the necessary tools to answer this question. *vsx1:GFP;ath5*$^{-/-}$*;ptf1a*$^{-/-}$*;ptf1aMOs* embryos were fixed at 72 hpf and stained with HuC/D to determine the extent of AC/RGC loss (Figures S2D and S2E, arrowheads). Despite the loss of all neuronal postsynaptic partners, the BC axons still appeared to organize into an actin-rich neuropil, or IPL-like neuropil, positioned along the basal surface of the retina (Figures 2C and 2D). The simultaneous elimination of RGCs and ACs did not reduce the width of the IPL appreciably compared with the elimination of ACs alone (Figure 2E). Interestingly, the IPL was significantly thicker than WT after the elimination of RGCs alone. Because ACs are overproduced in this context (Kay et al., 2001), the thickness of the IPL in zebrafish may largely reflect the contribution of AC processes.

The sublaminar organization of the IPL is proposed to result from homophilic adhesion and guidance cue and receptor interactions between pre- and postsyanaptic cell types (Matsuoka et al., 2011a, 2011b; Yamagata and Sanes, 2008, 2012; Yamagata et al., 2002). Therefore, we expected that IPL-like neuropil in the absence of ACs and RGCs might be completely devoid of any sublaminar organization. To test this, we used two transgenics that label different populations of BCs: *Q16* and *Q19*. *Q16 (nyx::mYFP)* labels a population of BCs that stratify in the basal half of the IPL (Schroeter et al., 2006). We generated a transgenic based on the *vsx1* promoter (*Q19*), which labels BCs that stratify in the apical half of the IPL. In WT retinas, imaging of the *Q16;Q19* double transgenic revealed two bands of nonoverlapping terminals in the IPL-like neuropil (Figures 2F and 2H). These double transgenics were then injected with the *ptf1aMOs* and *ath5MOs* to eliminate RGCs and many ACs. Although the sublamination of the IPL was clearly less organized than in the WT, in this very thin IPL-like neuropil the typical apical/basal pattern was still obvious (Figures 2G and 2I) and highly significant ($p = 3.0 \times 10^{-144}$; Figure 2J). Sublamination of BC populations was also observed

Cell PRESS
Open ACCESS

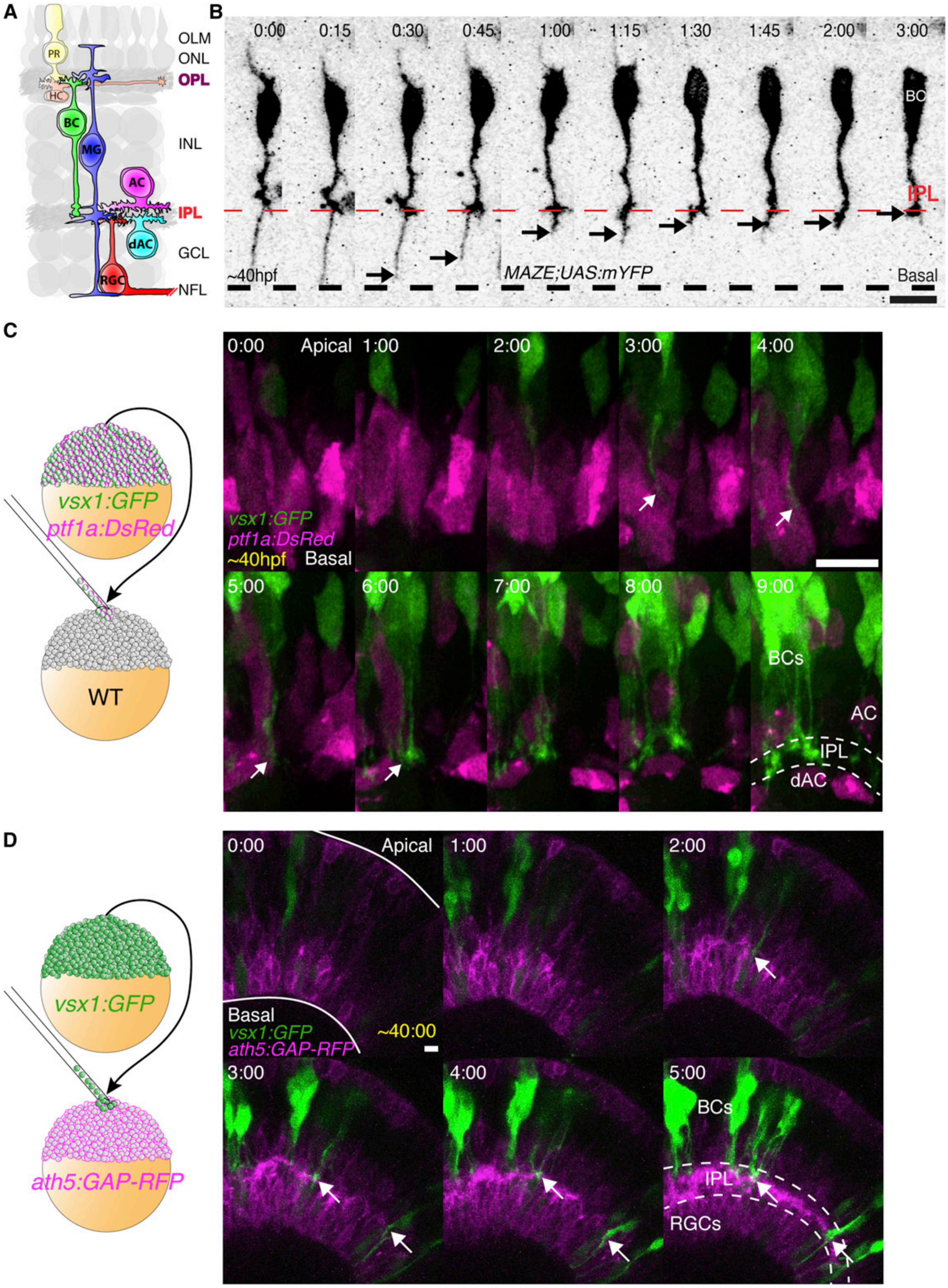

Figure 1. BC Axons Overshoot and Retract to Colonize the Nascent IPL

(A) Schematic showing the general organization of the vertebrate retina, including the neuropil layers, the IPL and OPL, and the retinal neurons and glia that will synapse within them.

(B) An individual BC labeled by *MAZe;UAS:mYFP* transgenes. The distal process extends to the basal surface (dashed line) of the retina. Branching into the IPL region can be seen, and the distal portion of the axon retracts to this point (arrow). At the same time, the apical process is also retracted from the apical surface of the retina.

(legend continued on next page)

using the markers *ath5:GAP-RFP* and protein kinase C (PKC) in *ath5−/−;ptf1a−/−;ptf1aMOs* embryos lacking ACs and RGCs (Figure S3).

We wondered whether BC axon terminals would differentiate properly in a neuropil lacking their normal postsynaptic partners. In the WT retina, the RibeyeA antibody labeled puncta in the IPL (Figure 3A), reflecting its accumulation in BC ribbon synapses. A similar pattern was seen in the AC/RGC-free neuropil (Figure 3B), indicating that presynaptic structures might still be forming. This was confirmed by electron microscopy (Figure 3C), which revealed vesicle-filled structures resembling BC axonal boutons in the neuropil. Surprisingly, ribbon structures with tethered vesicles in these BC boutons were sometimes localized at appositions with other BC boutons, almost as if they were trying to make synaptic contacts with each other (arrowhead, Figure 3C [inset] and s1–s3). However, because we did not find any postsynaptic densities at these membrane contact sites (arrowhead, Figures 3D and 3E), it is unlikely that these are functional synapses.

MG Are Not Required for IPL Formation

If BCs, ACs, and RGCs (i.e., the full neuronal complement of the IPL) are each unnecessary for the formation of synaptic neuropil, there is only one other cellular component of the IPL that could be essential: the MG. Indeed, in the zebrafish *mind bomb* (*mib*) mutant, which lacks MG, retinal layering fails (Bernardos et al., 2005). The early interference with Notch signaling in this mutant, however, appears to compromise the differentiation of many other retinal cell types. To remove MG more selectively, we took a pharmacological approach. We administered the Notch pathway-blocking gamma-secretase inhibitor N-[N-(3,5-difluorophenacetyl)-1-alanyl]-S-phenylglycine t-butyl ester (DAPT) at 30–33 hpf, after neurogenesis had begun. Using this treatment regime, we found that retinal layering was intact, the IPL and OPL formed, and all of the neuronal cell types were still present and correctly positioned. *vsx1:GFP*-labeled BCs and *ath5:GAP-RFP*-labeled RGCs, ACs, HCs, and PRs were all visible (Figure 4A). However, MG were completely absent throughout early development until at least 5 days postfertilization (dpf). This was confirmed by three separate immunohistochemical markers: anti-glutamine synthetase (anti-GS), anti-glial fibrillary acidic protein (anti-GFAP), and anti-cellular retinaldehyde-binding protein (anti-Cralbp; Figures 4B and 4C), as well as the transgenic marker *gfap:GFP* (Figure 4D). To determine whether the IPL was still properly organized into sublaminar compartments, we again made use of the *Q16* and *Q19* transgenes. After the MG were removed, the BC axon terminals still separated into their proper distinct layers (arrow, Figure 4D). We also assayed for the presence of synaptic proteins by staining for the presynaptic vesicle marker SV2, the ribbon synapse component RibeyeA, and the postsynaptic density protein Maguk. All were present after the removal of MG at 72 hpf, suggesting that synapses were still able to form (Figure 4E).

In the Absence of All Other Component Cells, BC Axons Still Make an IPL-Like Neuropil

We have shown that a presynaptic IPL-like neuropil still forms after the removal of ACs, RGCs, and MG. However, again considering the potential for redundancy in this system, it is possible that any one of these cell types is sufficient on its own, and only the simultaneous removal of all three cell types will prevent IPL formation. Therefore, we removed all three of these cell types simultaneously by treating *lak*$^{-/-}$*;ptf1a*$^{-/-}$*;ptf1aMO* embryos in DAPT beginning at 33 hpf. Although ACs, RGCs, and MG were all absent from these retinas (Figure 5A), the BC axons still formed an actin-rich, neuropil-like layer along the basal surface of the retina (arrowhead, Figure 5B), and contained the presynaptic protein RibeyeA (arrowhead, Figure 5C). Remarkably, this BC-axon-only IPL-like layer also maintained overall sublaminar structure between two BC populations (Figure 5D).

DISCUSSION

Previous work has shown that the IPL forms in the absence of either BCs or RGCs, pointing to either ACs or MG as being essential for retinal layering and IPL formation (Bernardos et al., 2005; Huberman et al., 2010; Kay et al., 2004; Willbold et al., 2000). Indeed, it was suggested that BCs are passive players in IPL formation and are recruited to the preformed IPL through interactions with partner neurons (Huberman et al., 2010; Kay et al., 2004). In contrast to these models, we find that BC axons stratify within the nascent IPL, and that neither ACs nor MG are essential for IPL-like neuropil formation. Not only are each of the individual cells discussed above unessential for IPL formation, but BCs are capable of forming a neuropil in a remarkably autonomous fashion when RGCs, ACs, and MG are simultaneously eliminated. Although BC axons are capable of forming a neuropil autonomously and are present within the nascent IPL, they are themselves dispensable for IPL formation in mice (Green et al., 2003; Tomita et al., 2000). It is also interesting to note that in the absence of RGCs and ACs, this IPL-like neuropil forms along the basal surface of the retina (rather than at its normal, more apical position), indicating that although these cells' partner cells may not be necessary for IPL formation, they may play a role in the positioning of this neuropil layer.

(C) Transplantation scheme to create mosaic retinas with clones of *vsx1:GFP*-expressing BCs and *ptf1a:DsRed*-expressing ACs in an unlabeled host retina. At the onset of imaging, *ptf1a:DsRed*-expressing ACs and HCs have migrated to the AC layer. However, a separation between the dACs and ACs is not apparent. Over time, the BC axons appear (arrows) and begin to stratify in between the *ptf1a:DsRed*-expressing cells. As these BC axons elaborate, the ACs are separated into displaced and nondisplaced populations that are parted by the expanding IPL. Images are confocal reconstructions.

(D) Transplantation scheme to create mosaic embryos with *vsx1:GFP*-expressing BCs in a host retina where RGCs and ACs are labeled by *ath5:GAP-RFP*. BC axons (arrows) accumulate coincidentally with the appearance of the IPL, as shown by *ath5:GAP-RFP*-labeled RGCs and ACs (dashed line). Images represent maximum intensity projections of nine confocal slices. Time shown in hr:min. Imaging begins at ~40 hpf. Scale bars = 10 μm. GCL, ganglion cell layer; INL, inner nuclear layer; NFL, neurofiber layer; OLM, outer limiting membrane; ONL, outer nuclear layer.

See also Figure S1 and Movies S1, S2, and S3.

Cell PRESS
Open ACCESS

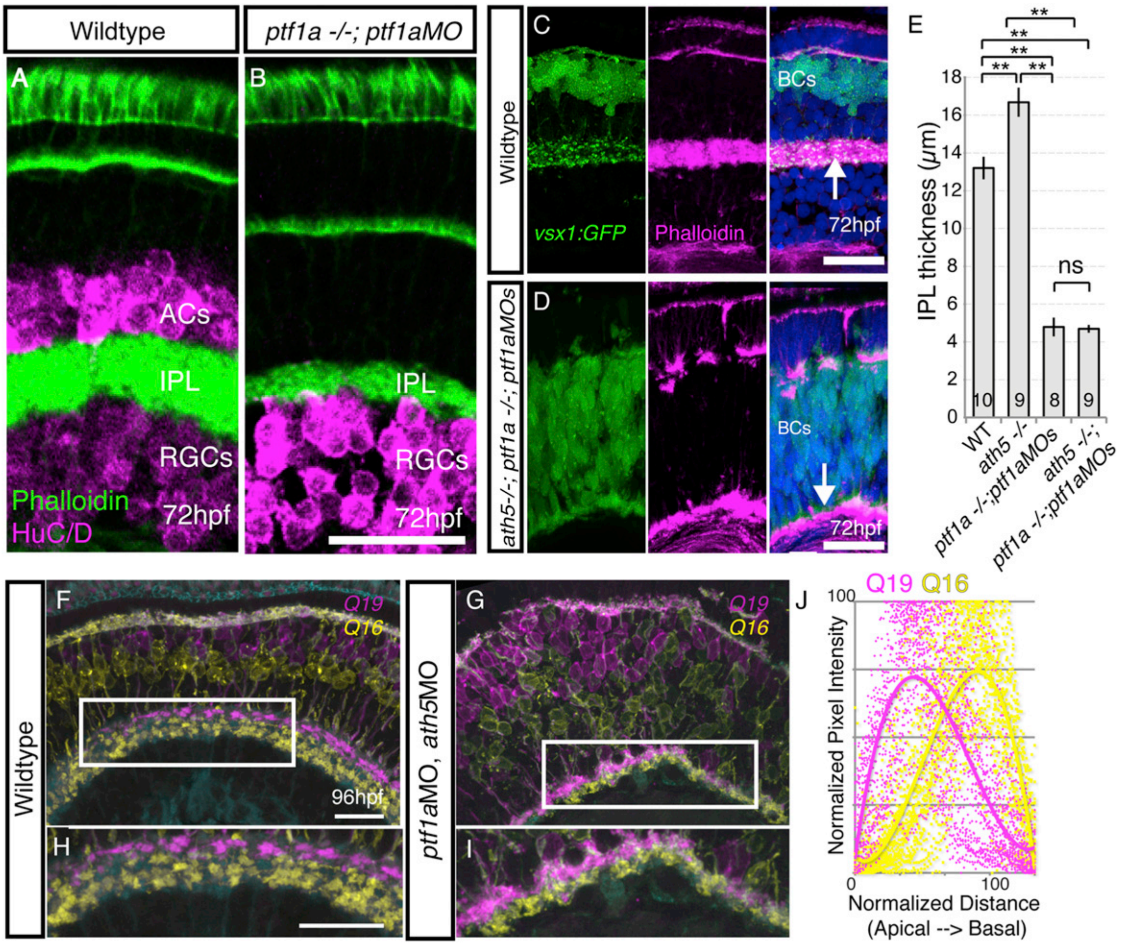

Figure 2. Simultaneous Removal of BC Partner Neurons (RGCs and ACs) Does Not Prevent BC Axons from Forming a Sublaminated Neuropil

(A and B) Despite the loss of ACs, a phalloidin-rich IPL forms in the *ptf1a* $^{-/-}$;*ptf1aMO* retina.

(C) WT retina with the actin-rich IPL shown by phalloidin (arrow) and BC terminals labeled with *vsx1:GFP*.

(D) Phalloidin staining demonstrates that in the AC/RGC-free retina (*ath5*$^{-/-}$;*ptf1a*$^{-/-}$;*ptf1aMO*), the IPL is actin rich and positioned along the basal surface of the retina (arrow).

(E) The thickness of the IPL was measured for each genotype at 5 dpf, demonstrating it is significantly thinner after AC removal (*ptf1a*$^{-/-}$;*ptf1aMOs*), and thicker after RGC removal (*ath5*$^{-/-}$). One-way ANOVA and Tukey post hoc tests; $^{**}p < 0.01$; error bars ± 1 SEM. The number of retinas measured is given within the bars.

(F) In the WT retina, *Q16*-labeled BCs stratify basally to *Q19*-labeled BCs in the IPL.

(G) After the removal of many ACs and RGCs by *ptf1aMO* and *ath5MO* injections (G), the typical *Q16*-basal, *Q19*-apical pattern is apparent in many areas.

(H) Inset in (F).

(I) Inset in (G).

(J) Line intensity profile measurements from 45 regions (three different retinas/animals) demonstrates the basal enrichment of *Q16* signal and apical enrichment of *Q19* signal in *ath5;ptf1a* morphants. Scale bars, 20 μm.

See also Figures S2 and S3.

It is important to note that our treatment using mutants, morpholinos, and/or pharmacological inhibitors does not result in a loss of these cells from the retina; rather, the cells are respecified to other available fates. Although it is unlikely, we cannot rule out the possibility that respecified cells retain properties reminiscent of their original fate (e.g., ACs with some RGC properties in *ath5*$^{-/-}$) that may subsequently affect lamination and/or BC axon positioning. Similarly, we were not able to completely rid the retina of ACs, but it seems very unlikely that the <1% of ACs that remain can drive the stratification of all other neurons across the retina.

Interestingly, in the absence of normal postsynaptic partners, BCs still appear to form presynaptic terminals replete with ribbons and synaptic vesicles abutting the processes of other BCs. This is reminiscent of previous reports that proper postsynaptic muscle targets are not necessary for presynaptic specializations in the *Drosophila* neuromuscular junction (Prokop et al., 1996), and that cultured spinal cord axons will form presynaptic specializations when in contact with a polyornithine-coated bead (Peng et al., 1987). More surprisingly, the BC-only neuropil exhibited clear vestiges of sublaminar organization. BC axons that normally laminate in the apical half of the IPL still laminated

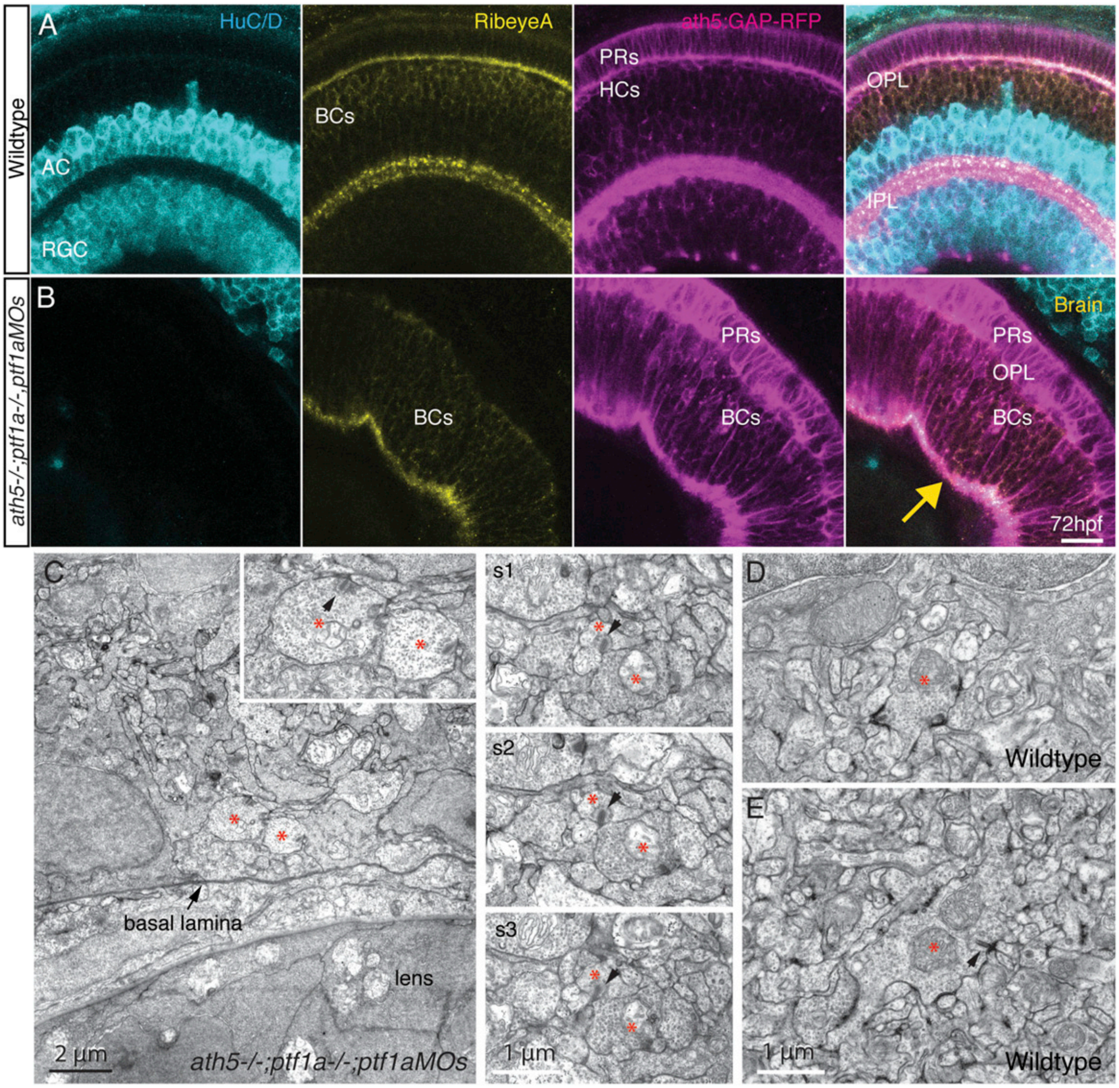

Figure 3. BC Axons Form Presynaptic Structures without Postsynaptic Neurons

(A) WT *ath5:GAP-RFP* retinas stained with anti-HuC/D (to label ACs/RGCs) and anti-RibeyeA (to label ribbon synapses). Obvious punctate RibeyeA staining is visible in the IPL, whereas weak staining is seen in the BC cell bodies in the apical half of the INL and the OPL.

(B) When ACs and RGCs are absent in the *ath5:GAP-RFP*;*ath5*$^{-/-}$;*ptf1a*$^{-/-}$;*ptf1aMO* embryos, the weakly RibeyeA-staining BC cell bodies span the entire INL, and punctate RibeyeA staining is visible in the BC-only IPL. Scale bar in (A) and (B), 10 μm.

(C) Examples of vesicle-filled bouton-like structures (asterisks) containing ribbons near the retinal basal lamina of animals with severely reduced numbers of ACs and RGCs. Left: Higher magnification of the two such structures, presumed to be BC axonal boutons (arrowhead) shown in the inset. Right: Three consecutive sections (s1–s3) of processes in basally located neuropil in another animal. Arrows indicate ribbons juxtaposed to appositions with other processes, one of which can be another BC terminal (s1–s3).

(D and E) WT BC synapses exhibit readily apparent postsynaptic densities (thickenings, arrow) that are not seen in the mutant/morphant plexus (s1–s3).

apically to BC axons, which normally laminate basally. This observation highlights the importance of interactions among classes of presynaptic neurons, rather than simply among different types of neurons and glia, in setting up these segregated layers.

Because no single intrinsic retinal cell type is absolutely essential for retinal neuropil formation, and BCs can form a rudimentary neuropil autonomously, it seems reasonable to suspect that each of the three major neuronal types that contribute neurites to the IPL may also be able to do so autonomously. This is in line with studies in the embryonic *Drosophila* nerve cord, wherein it has been suggested that growing axons and dendrites are independently delivered to appropriate volumes of the developing neuropil by position-dependent guidance cues (Zlatic et al., 2009). If this explanation is correct, it will be important to discover which guidance cues are used to establish and position the retinal neuropil layers and how these cues are regulated.

Cell PRESS
Open ACCESS

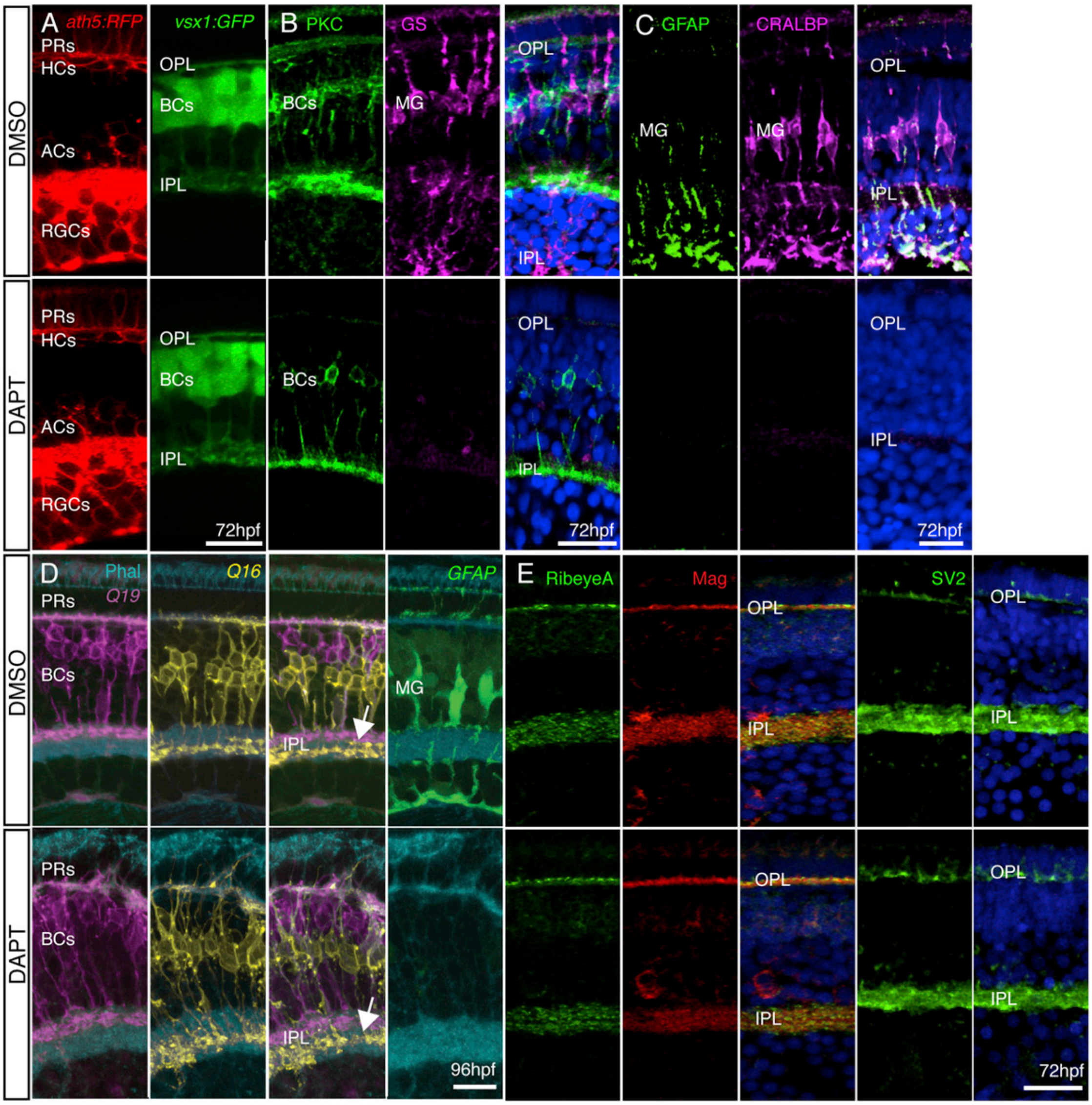

Figure 4. MG Are Not Required for Retinal Organization or Plexiform Layer Development

(A) *ath5:GAP-RFP* and *vsx1:GFP* show that all neural retinal cell types are present and correctly positioned in DMSO-treated control embryos and after DAPT treatment.

(B and C) Treatment with DAPT at 33 hpf completely removed MG in the retina. PKC staining is unperturbed by DAPT treatment, indicating BC differentiation is unaffected. However, DAPT-treated embryos show a complete loss of MG when stained with specific markers for GS, CRALBP, and GFAP when compared with DMSO controls.

(D) The IPL forms and sublaminates properly in the absence of MG. *nyx:Gal4; UAS:MYFP*^{Q16}, and *vsx1:MCerulean*^{Q19} fish show labeling of specific subsets of BCs that stratify in the apical (OFF) or basal (ON) domains of the IPL. Imaging of triple transgenics *Q16;Q19;gfap:GFP* treated with DAPT shows that the IPL still forms and BC axons sublaminate properly (arrow). Lack of *gfap:GFP* signal confirms that there are no MG in the areas of proper sublamination.

(E) Pre- and postsynaptic markers remain in the plexiform layers lacking MG. Staining for the presynaptic markers RibeyeA and SV2 and the postsynaptic density protein Maguk (Mag) is unperturbed in embryos lacking MG at 72 hpf. Scale bars, 10 μm (A and D) and 20 μm (B, C, and E).

EXPERIMENTAL PROCEDURES

Transgenic Lines and Constructs

Transgenic lines Tg*(atoh7:gap43-mRFP1)cu2*, *Tg(vsx1:GFP)nns5*, *Tg(MAZe)*, *Tg(Ptf1a:DsRed)*, and *Tg(nyx:Gal4-VP16)q16a;Tg(UAS:gap43-YFP)q16b* have been described previously (Collins et al., 2010; Kimura et al., 2008; Schroeter et al., 2006; Vitorino et al., 2009; Zolessi et al., 2006) and are abbreviated here as *ath5:GAP-RFP*, *vsx1:GFP*, *MAZe*, *ptf:DsRed*, and *Q16*, respectively. The *Vsx1:MCerulean* (Q19) line was created using the upstream region of the Vsx1 gene (see Extended Experimental Procedures).

DAPT Treatment

Zebrafish embryos were treated with DAPT (50 μM) in 0.5% DMSO solution from 33 hpf to 3–4 dpf, and then fixed with 4% paraformaldehyde and cryoprotected in 30% sucrose in 1X PBS overnight prior to cryosectioning.

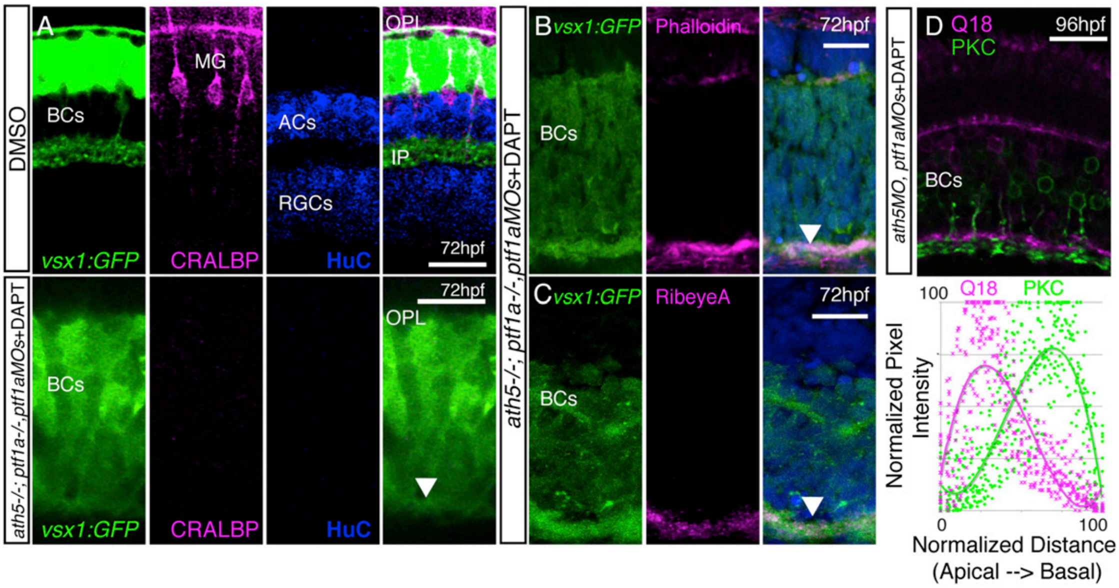

Figure 5. BC Axons Can Form a Sublaminated Neuropil in the Absence of Partner Neurons and Glia

(A–C) A neuropil layer (arrowhead) forms at the basal surface of a cellularly simplified retina *ath5*$^{-/-}$*;ptf1a*$^{-/-}$*;ptf1aMOs* treated with DAPT, lacking ACs, HCs, RGCs, and MG (A). The neuropil layer is actin rich (B) and contains RibeyeA positive staining at 72 hpf (C).

(D) Gross sublaminar structure is maintained in cellularly simplified retinas, with Q19 apical to the PKC staining in the IPL-like layer. This sublamination was statistically significant ($p = 6.22 \times 10^{-44}$, n = 3 sections). Scale bars, 10 μm (A, D, and E) and 20 μm (B, C, F, G, H, and I).

SUPPLEMENTAL INFORMATION

Supplemental Information includes three figures, four movies, and Extended Experimental Procedures and can be found with this article online at http://dx.doi.org/10.1016/j.celrep.2013.01.020.

LICENSING INFORMATION

This is an open-access article distributed under the terms of the Creative Commons Attribution License, which permits unrestricted use, distribution, and reproduction in any medium, provided the original author and source are credited.

ACKNOWLEDGMENTS

We thank M. Agathocleous for suggesting the Notch inactivation experiments; C. O'Hare and H. Boije for critical readings of the manuscript; A. McNabb, T. Dyl, K.L. Scott, and S. Waldron for fish maintenance; and J. He, C. Norden, P. Jusuf, and members of the Wong, Harris, and Holt laboratories for helpful advice and discussions. We also thank K. Verhey for the Kif5c-mCherry construct, T. Nicholson for the RibeyeA antibody, J. Saari for the Cralbp antibody, J. Fadool for the 5E11 antibody, S. Higashijima for the *vsx1:GFP* transgenic, S. Leach for the *ptf1a:DsRed* transgenic, and the Wellcome Trust Sanger Institute Zebrafish Mutation Project for the *ptf1a* mutant line. We thank E. Parker for technical assistance with electron microscopy. This work was funded by grants from the Wellcome Trust (to W.A.H.), the NIH (EY14358 to R.O.W. and Vision Core grant EY01730 to the University of Washington), and the Wellcome Trust Programme in Developmental Biology and the Cambridge Overseas Trust (O.R.).

Received: August 3, 2012
Revised: November 21, 2012
Accepted: January 16, 2013
Published: February 14, 2013

REFERENCES

Bernardos, R.L., Lentz, S.I., Wolfe, M.S., and Raymond, P.A. (2005). Notch-Delta signaling is required for spatial patterning and Müller glia differentiation in the zebrafish retina. Dev. Biol. *278*, 381–395.

Collins, R.T., Linker, C., and Lewis, J. (2010). MAZe: a tool for mosaic analysis of gene function in zebrafish. Nat. Methods *7*, 219–223.

Deans, M.R., Krol, A., Abraira, V.E., Copley, C.O., Tucker, A.F., and Goodrich, L.V. (2011). Control of neuronal morphology by the atypical cadherin Fat3. Neuron *71*, 820–832.

Distel, M., Hocking, J.C., Volkmann, K., and Köster, R.W. (2010). The centrosome neither persistently leads migration nor determines the site of axonogenesis in migrating neurons in vivo. J. Cell Biol. *191*, 875–890.

Godinho, L., Mumm, J.S., Williams, P.R., Schroeter, E.H., Koerber, A., Park, S.W., Leach, S.D., and Wong, R.O. (2005). Targeting of amacrine cell neurites to appropriate synaptic laminae in the developing zebrafish retina. Development *132*, 5069–5079.

Green, E.S., Stubbs, J.L., and Levine, E.M. (2003). Genetic rescue of cell number in a mouse model of microphthalmia: interactions between Chx10 and G1-phase cell cycle regulators. Development *130*, 539–552.

Huberman, A.D., Clandinin, T.R., and Baier, H. (2010). Molecular and cellular mechanisms of lamina-specific axon targeting. Cold Spring Harb. Perspect. Biol. *2*, a001743.

Jacobson, C., Schnapp, B., and Banker, G.A. (2006). A change in the selective translocation of the Kinesin-1 motor domain marks the initial specification of the axon. Neuron *49*, 797–804.

Jusuf, P.R., and Harris, W.A. (2009). Ptf1a is expressed transiently in all types of amacrine cells in the embryonic zebrafish retina. Neural Dev. *4*, 34.

Jusuf, P.R., Almeida, A.D., Randlett, O., Joubin, K., Poggi, L., and Harris, W.A. (2011). Origin and determination of inhibitory cell lineages in the vertebrate retina. J. Neurosci. *31*, 2549–2562.

Kay, J.N., Finger-Baier, K.C., Roeser, T., Staub, W., and Baier, H. (2001). Retinal ganglion cell genesis requires lakritz, a Zebrafish atonal Homolog. Neuron *30*, 725–736.

Kay, J.N., Roeser, T., Mumm, J.S., Godinho, L., Mrejeru, A., Wong, R.O., and Baier, H. (2004). Transient requirement for ganglion cells during assembly of retinal synaptic layers. Development *131*, 1331–1342.

Kimura, Y., Satou, C., and Higashijima, S. (2008). V2a and V2b neurons are generated by the final divisions of pair-producing progenitors in the zebrafish spinal cord. Development *135*, 3001–3005.

Layer, P.G., Rothermel, A., and Willbold, E. (1998). Inductive effects of the retinal pigmented epithelium (RPE) on histogenesis of the avian retina as revealed by retinospheroid technology. Semin. Cell Dev. Biol. *9*, 257–262.

Matsuoka, R.L., Chivatakarn, O., Badea, T.C., Samuels, I.S., Cahill, H., Katayama, K., Kumar, S.R., Suto, F., Chédotal, A., Peachey, N.S., et al. (2011a). Class 5 transmembrane semaphorins control selective Mammalian retinal lamination and function. Neuron *71*, 460–473.

Matsuoka, R.L., Nguyen-Ba-Charvet, K.T., Parray, A., Badea, T.C., Chédotal, A., and Kolodkin, A.L. (2011b). Transmembrane semaphorin signalling controls laminar stratification in the mammalian retina. Nature *470*, 259–263.

Morgan, J.L., Dhingra, A., Vardi, N., and Wong, R.O. (2006). Axons and dendrites originate from neuroepithelial-like processes of retinal bipolar cells. Nat. Neurosci. *9*, 85–92.

Peng, H.B., Markey, D.R., Muhlach, W.L., and Pollack, E.D. (1987). Development of presynaptic specializations induced by basic polypeptide-coated latex beads in spinal cord cultures. Synapse *1*, 10–19.

Prokop, A., Landgraf, M., Rushton, E., Broadie, K., and Bate, M. (1996). Presynaptic development at the Drosophila neuromuscular junction: assembly and localization of presynaptic active zones. Neuron *17*, 617–626.

Randlett, O., Poggi, L., Zolessi, F.R., and Harris, W.A. (2011). The oriented emergence of axons from retinal ganglion cells is directed by laminin contact in vivo. Neuron *70*, 266–280.

Robu, M.E., Larson, J.D., Nasevicius, A., Beiraghi, S., Brenner, C., Farber, S.A., and Ekker, S.C. (2007). p53 activation by knockdown technologies. PLoS Genet. *3*, e78.

Schroeter, E.H., Wong, R.O., and Gregg, R.G. (2006). In vivo development of retinal ON-bipolar cell axonal terminals visualized in nyx:MYFP transgenic zebrafish. Vis. Neurosci. *23*, 833–843.

Timofeev, K., Joly, W., Hadjieconomou, D., and Salecker, I. (2012). Localized netrins act as positional cues to control layer-specific targeting of photoreceptor axons in Drosophila. Neuron *75*, 80–93.

Tomita, K., Moriyoshi, K., Nakanishi, S., Guillemot, F., and Kageyama, R. (2000). Mammalian achaete-scute and atonal homologs regulate neuronal versus glial fate determination in the central nervous system. EMBO J. *19*, 5460–5472.

Vitorino, M., Jusuf, P.R., Maurus, D., Kimura, Y., Higashijima, S., and Harris, W.A. (2009). Vsx2 in the zebrafish retina: restricted lineages through derepression. Neural Dev. *4*, 14.

Willbold, E., Rothermel, A., Tomlinson, S., and Layer, P.G. (2000). Müller glia cells reorganize reaggregating chicken retinal cells into correctly laminated in vitro retinae. Glia *29*, 45–57.

Yamagata, M., and Sanes, J.R. (2008). Dscam and Sidekick proteins direct lamina-specific synaptic connections in vertebrate retina. Nature *451*, 465–469.

Yamagata, M., and Sanes, J.R. (2012). Expanding the Ig superfamily code for laminar specificity in retina: expression and role of contactins. J. Neurosci. *32*, 14402–14414.

Yamagata, M., Weiner, J.A., and Sanes, J.R. (2002). Sidekicks: synaptic adhesion molecules that promote lamina-specific connectivity in the retina. Cell *110*, 649–660.

Zlatic, M., Li, F., Strigini, M., Grueber, W., and Bate, M. (2009). Positional cues in the Drosophila nerve cord: semaphorins pattern the dorso-ventral axis. PLoS Biol. *7*, e1000135.

Zolessi, F.R., Poggi, L., Wilkinson, C.J., Chien, C.B., and Harris, W.A. (2006). Polarization and orientation of retinal ganglion cells in vivo. Neural Dev. *1*, 2.

Supplemental Information

Cell PRESS Open ACCESS

EXTENDED EXPERIMENTAL PROCEDURES

Animals

Adult zebrafish were maintained and bred at 26.5°C. Embryos were raised at 25°C–32°C and staged based on hpf (Kimmel et al., 1995). Embryos were treated with 0.003% phenylthiourea (Sigma) from 10 hpf to prevent pigmentation. All animal work was approved by Local Ethical Review Committee at the University of Cambridge and performed according to the protocols of project license PPL 80/2198 and according to institutional guidelines (IACUC) of the University of Washington.

Creation of Plasmids and Transgenic Lines

A pTol2CG2-Vsx1:Mcerulean plasmid was made using a tol2kit (Kwan et al., 2007). A 3.2 kb Vsx1 gene promoter region was amplified from the BAC plasmid containing zebrafish vsx1 gene region by PCR using primers; 5′-GGGGACAACTTTGTATAGAAAAGTTGG CAGTCAGTCAGCCCTTCTC-3′ and 5′-GGGGACTGCTTTTTTGTACAAACTTGATTGTCGATTCCGAACGAAGGGTA-3′, to make p5E-Vsx1 plasmid. This plasmid was recombined into pTol2CG2 plasmid together with pME-MCerulean and p3E-pA plasmids using GATEWAY system (Invitrogen).

The PCS2:Kif5c-mCherry construct was created by PCR amplification of the Kif5c560-mcherry ORF from pBactKif5c560-mCherry (Hammond et al., 2010) using the following primers (frw_5′-GGGGAATTCATGGCGGATCCAGCCGAATG-3′ and rev_5′-CCCTCTAG ATTACTTGTACAGCTCGTCCATGCCG-3′) and was subcloned into the EcoR1 and Xba1 sites of PCS2+. mRNA for injection was created by linearizing with Not1 enzyme and synthesizing capped RNA from the Sp6 promoter using mMessage Machine SP6 Kit (Ambion).

To label individual BCs we crossed the MAZe (Collins et al., 2010) transgenic fish to a UAS:mYFP reporter line (Williams et al., 2010). In MAZe fish, a heat shock is used to drive Cre recombinase allowing expression of Gal4/UAS expression. Heat shock was applied at 30 hpf for 2 min at 39°C. Embryos were screened under a fluorescent microscope for individually labeled BC cells within the retina at 36hpf and imaged using the extended imaging protocols (described below).

Data Analysis

Confocal data was analyzed and processed using Volocity (Improvision), ImageJ/FIJI (NIH), and Amira (Visage Imaging, Andover, Massachusetts, USA). Deconvolution was generally performed on data acquired by spinning disk confocal microscopy using the Iterative Restoration tool at 25 iterations and 99.99% confidence levels. Intensity profile measurements were done using the “plot profile” tool in ImageJ, using a line with a 20 or 30-pixel width. To plot all line profiles on a single graph, fluorescent intensity was normalized to the maximum and minimum value for each line profile, and was normalized for relative position along the line. Third order polynomial best-fit lines were drawn using Excel (Microsoft). Length measurements were done using the line tool in Volocity. Statistical tests were performed using Instat (GraphPad), Matlab (Mathwords) and Excel. To test for significance in sublaminar sorting (for example Q16 versus Q19 signal), we asked at which normalized positions along the IPL width was the signal from a given channel above a threshold (50% of the maximal signal observed along that line). These position distributions were tested for statistical significance (Mann-Whitney U-test). In cases where fluorescent signal was not measured quantitatively the brightness, contrast, and gamma of images was adjusted for maximal visibility of cellular morphology and fluorescent signal using Volocity, Photoshop (Adobe), and ImageJ. Some confocal images were median-filtered to reduce noise. Vertical sections of the *Q16;Q19* retinas were viewed by rotating the image stack and digitally sectioning the imaged volume at a plane that is parallel to the apical-basal axis, using the Orthoslice function of AMIRA.

Embryo Manipulations

RNA and morpholinos were injected into the yolk of one- cell stage embryos. A mixture of 12ng *ptf1a* MO1 (5′-CCAACACAGTGTCC ATTTTTTGTGC-3′, Gene Tools), *ptf1a* MO4 (5′-TTGCCCAGTAACAACAATCGCCTAC-3′, Gene Tools) and *p53* MO (5′- GCGC CATTGCTTTGCAAGAATTG-3′, Gene Tools) was injected to prevent AC formation (Jusuf et al., 2011). 4ng of *ath5* MO (5′TTCA TGGCTCTTCAAAAAAGTCTCC-3′, Gene Tools) was injected to prevent RGC formation (Pittman et al., 2008).

For blastomere transplantations, high- to oblong-stage embryos were dechorionated by pronase digestion (Sigma), placed in agarose molds, and between 5 and 30 blastomeres were transferred between embryos using a glass capillary connected to a 2 ml syringe. For transplants from Kif5c560-mCherry expressing donors, the p53 morpholino was injected into donor embryos to prevent apoptosis as this construct exhibited a mild degree of cellular toxicity.

Microscopy

Confocal imaging of live and fixed embryos was performed as described previously (Das et al., 2003). For TEM, larvae were fixed with 4% glutaraldehyde in 0.1 M sodium cacodylate buffer, pH 7.4 for several hours. After washing in buffer, they were postfixed in1% OsO_4 in cacodylate buffer and stained en bloc with 1% uranyl acetate. After immersion in a graded ethanol series, the animals were embedded in Araldite, sectioned and stained with 1% lead citrate prior to viewing.

Imaging and Immunostaining

For *Q16;Q19* transgene imaging, embryos were fixed at 96 hpf with 4% paraformaldehyde in 2% sucrose and 0.1 M PBS for 1 hr. Fish were enucleated and the eyes incubated in a blocking solution (5% normal goat serum and 0.5% Triton X-100 containing 0.1M PBS)

for 1 hr, stained with Alexa Fluor 633 conjugated Phalloidin (invitrogen) in this solution for 1 hr and then washed in 0.1 M PBS. After washing, the eyes were hemisected, mounted into 0.7% agar and coverslipped in Vectashield (Vector Laboratories).

Spinning disk confocal imaging was performed using a Perkin Elmer Spinning Disk UltraVIEW ERS, Olympus IX81 Inverted microscope and 60 × (1.2 NA) water immersion objective. Laser scanning confocal imaging was performed using an Olympus FV1000 microscope with a 60 × silicone immersion objective (1.3 NA) or a 60 x oil objective (1.35 NA), or with an SP2 microscope (Leica) with a 63 × (1.2 NA) water immersion objective. For live imaging, optical sections at 0.5–1 μm separation were taken to cover the region of retina containing the cells of interest (between 40 and 100 μm), A motorized XY stage (H117, Prior) was used to image multiple embryos simultaneously.

Immunostaining was performed using standard methods, using alexa-conjugated secondary antibodies (Invitrogen) and the following primary antibodies: mouse anti-HuC/D (1:200, 16A11, Invitrogen), mouse anti-5E11 (1:100, a gift from J. Fadool), mouse anti-panMaguk (1:100, clone K28/86, Neuromab), mouse anti-SV2 (1:100, Developmental Studies Hybridoma Bank), rabbit anti-PKCβ1 (1:150, sc-209, SantaCruz), mouse anti-Glutamine Synthase (GS; 1:50, mab302, Millipore), rabbit anti-Cralbp (1:1000 a gift from J. Saari), mouse anti-GFAP (1:100 zrf1, ZIRC), phalloidin-alexa488 (1:50 Invitrogen) and rabbit anti-ribeyeA (1:1000 and gift from Teresa Nicholson). Cryosectections were taken at 12-20 μm thickness using a Jung Frigocut cryostat (Leica).

SUPPLEMENTAL REFERENCES

Das, T., Payer, B., Cayouette, M., and Harris, W.A. (2003). In vivo time-lapse imaging of cell divisions during neurogenesis in the developing zebrafish retina. Neuron *37*, 597–609.

Hammond, J.W., Huang, C.F., Kaech, S., Jacobson, C., Banker, G., and Verhey, K.J. (2010). Posttranslational modifications of tubulin and the polarized transport of kinesin-1 in neurons. Mol. Biol. Cell *21*, 572–583.

Kimmel, C.B., Ballard, W.W., Kimmel, S.R., Ullmann, B., and Schilling, T.F. (1995). Stages of embryonic development of the zebrafish. Dev. Dyn. *203*, 253–310.

Kwan, K.M., Fujimoto, E., Grabher, C., Mangum, B.D., Hardy, M.E., Campbell, D.S., Parant, J.M., Yost, H.J., Kanki, J.P., and Chien, C.B. (2007). The Tol2kit: a multisite gateway-based construction kit for Tol2 transposon transgenesis constructs. Dev. Dyn. *236*, 3088–3099.

Pittman, A.J., Law, M.Y., and Chien, C.B. (2008). Pathfinding in a large vertebrate axon tract: isotypic interactions guide retinotectal axons at multiple choice points. Development *135*, 2865–2871.

Williams, P.R., Suzuki, S.C., Yoshimatsu, T., Lawrence, O.T., Waldron, S.J., Parsons, M.J., Nonet, M.L., and Wong, R.O. (2010). In vivo development of outer retinal synapses in the absence of glial contact. J. Neurosci. *30*, 11951–11961.

Cell PRESS
Open ACCESS

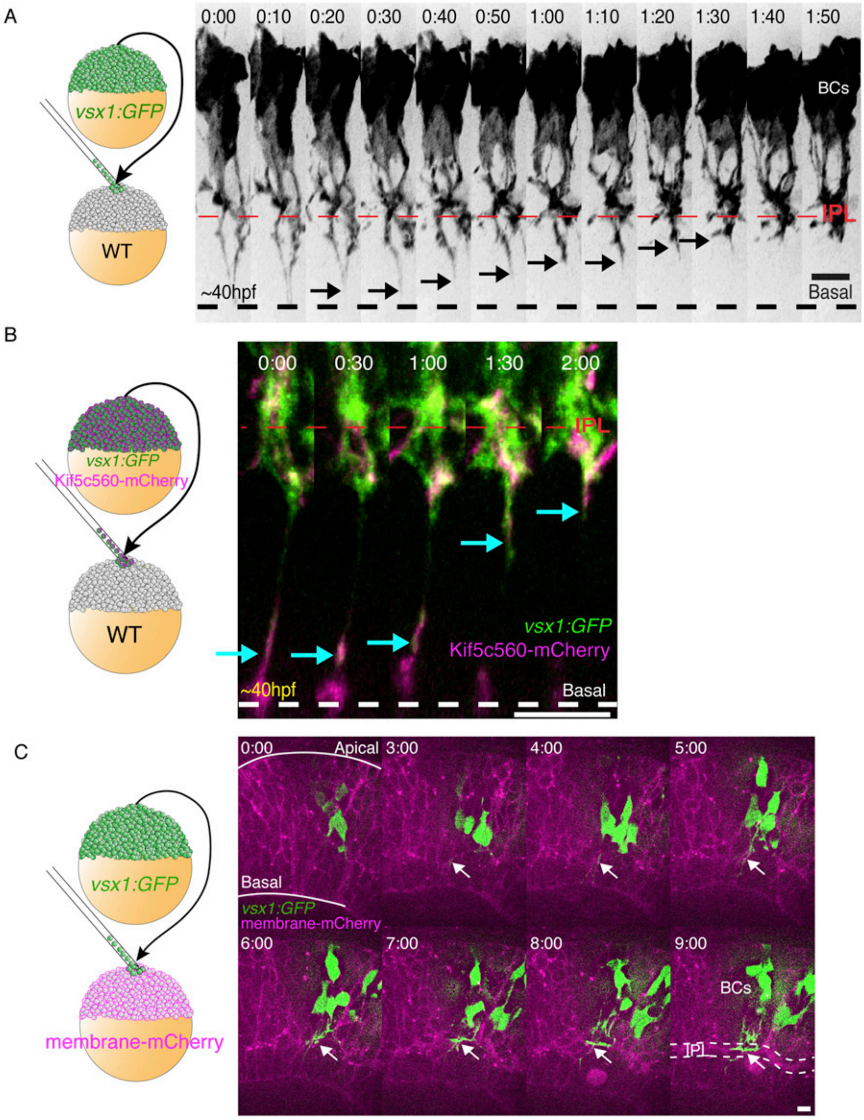

Figure S1. BC Axons Colonize the Nascent IPL at Approximately the Same Time as Other Retinal Cell Types, Related to Figure 1

(A) Transplantation scheme to create mosaic embryos with vsx1:GFP-labeled BCs in a WT host. BCs initially display a long process extending to the basal surface of the retina (dashed line). As development progresses, the distal portions of the basal processes retract (arrowheads) and the BC axons collect, condense and branch directly into the IPL.

(B) Transplantation scheme to create mosaic embryos mosaic embryos with Kif5c560-mCherry and vsx1:GFP co-expressing BCs within a WT host retina. We observe bright Kif5c560-mCherry signal visible within retracting BC process (arrows). Time given in hr:min. Time-lapse imaging begins at 40-45hpf.

(C) Transplantation scheme to create mosaic retinas with clones of *vsx1:GFP* expressing BCs within a membrane-mCherry labeled retina. The IPL becomes visible in the mCherry channel as a wavy band (t = 3:00), which thickens and condenses into the IPL (dotted line, t = 9:00). The GFP-expressing BC axons are visible within the nascent IPL (arrows), and elaborate as the IPL matures. Scale bars = 10 μm.

Cell PRESS
Open ACCESS

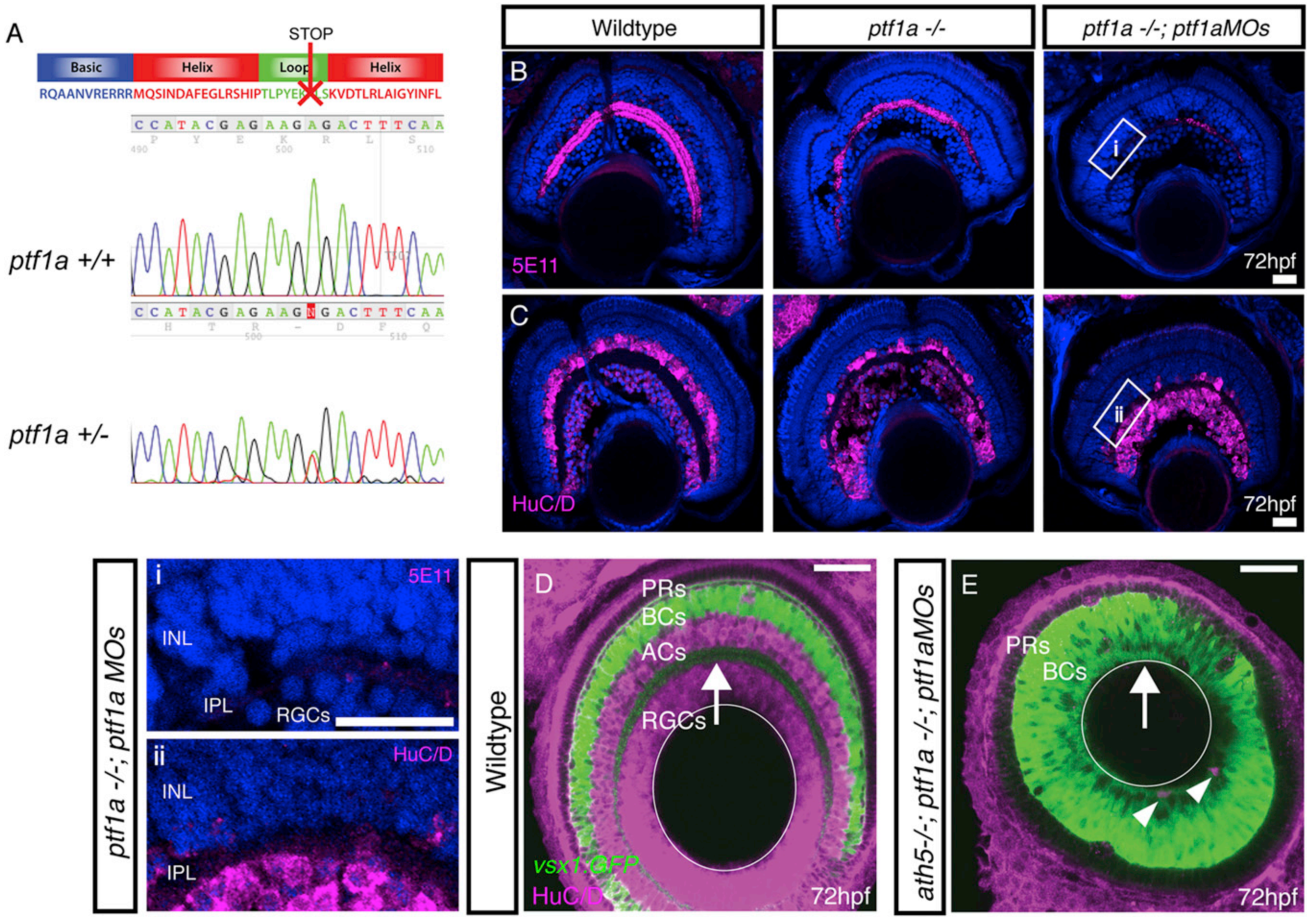

Figure S2. Elimination of ACs through Ptf1a Disruption, Related to Figure 2

(A) The *ptf1a* ^{sa126} mutation is an A to T conversion, which results in a non-sense mutation at amino acid 126 of 265. This results in a truncation within the loop of the basic helix-loop-helix DNA binding domain.

(B and C) Immunostaining of retinal cryosections using (B) 5E11 to stain AC neurites and (C) anti-HuC/D to stain RGC and AC cell bodies, indicates that some ACs remain in homozygous *ptf1a* $^{-/-}$ mutants. Injection of *ptf1a* morpholinos into *ptf1a*$^{-/-}$ mutants, causes a further reduction in ACs numbers, where large stretches of retina lack 5E11 staining and HuC/D positive cells in the AC layer (higher magnification inserts given in: i, and ii).

(D) WT retina with the vsx1GFP labeling BC axons in the IPL (arrow) and ACs/RGCs labeled with HuC/D.

(E) In the *ath5*$^{-/-}$;*ptf1a*$^{-/-}$;*ptf1aMO*, AC/RGC-free retina, the IPL is positioned along the basal surface of the retina (arrow). Few remaining ACs shown with arrowheads.

Cell PRESS
Open ACCESS

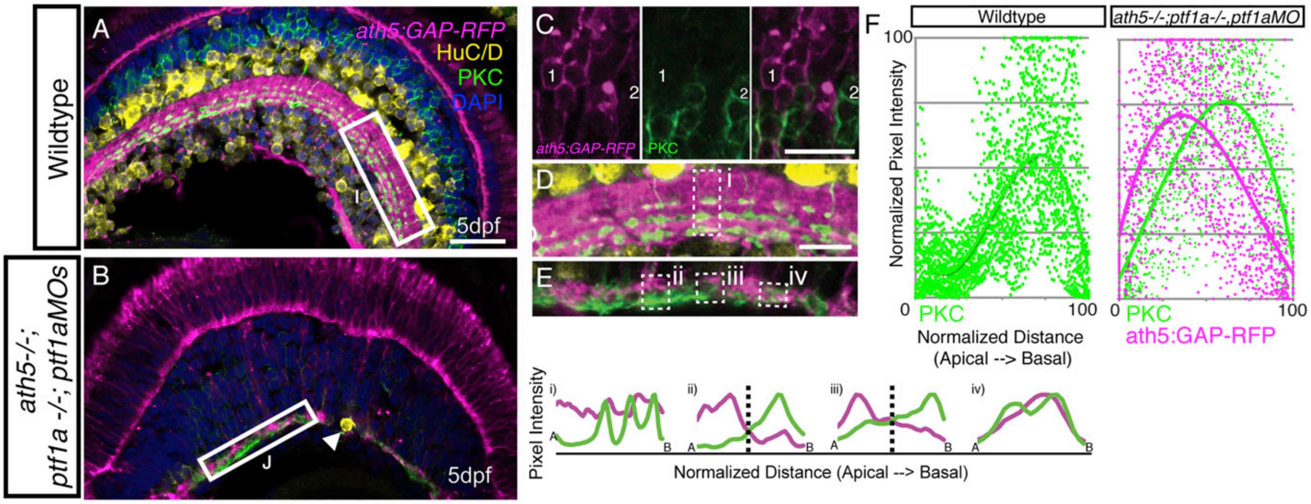

Figure S3. BC Axons Can Form a Sublaminated Neuropil in the Absence of Partner Neurons, Related to Figure 2

(A) Cryosections from 5 dpf *ath5:GAP-RFP* transgenic stained with anti-PKC (labeling ON-BCs) and HuC/D (labeling RGCs and ACs).

(B) *ath5*$^{-/-}$*ptf1a*$^{-/-}$*;ptf1a MO* retinas lack HuC/D positive RGCs and ACs (remaining AC - arrowhead), but an *ath5:GAP-RFP*/PKC-labeled IPL still forms.

(C) In the *ath5:GAP-RFP;ath5*$^{-/-}$*ptf1a*$^{-/-}$*;ptf1a MO* retinas, the ath5:GAP-RFP label is expressed by a subpopulation of BCs in the INL, which are distinct from the population of BCs labeled by the anti-PKC (cell #1 is *ath5:GAP-RFP*-positve, while cell #2 is PKC-positive).

(D) Higher magnification inset of the WT IPL showing the targeting of PKC axon terminals to 3 sublaminae in the basal half of the IPL. This is shown quantitatively by three peaks in the line intensity profile in region (i).

(E) The AC/RGC-free IPL shows some degree of disorganization. Yet, in some areas the PKC signal clearly segregates basal to the ath5:GAP-RFP signal in a stratified pattern (ii and iii). In other areas this is not the case (iv).

(F) Line intensity profiles were normalized for intensity of relative apical/basal position along the IPL, and plotted as a single graph. This demonstrates the basal accumulation of PKC-labeled axons typical of the WT retina (n = 30 measurements, from 10 sections), as well and the basal enrichment of the PKC signal and the apical enrichment of the ath5:GAP-RFP signal in the *ath5:GAP-RFP;ath5*$^{-/-}$*ptf1a*$^{-/-}$*;ptf1a MO* retinas (n = 54 measurements, from 18 sections), are is reflected in the shift of the trendline. Note that ath5:GAP-RFP signal quantification is not shown for WT, as this transgene is expressed by RGCs and ACs in this context.
